# Multidisciplinary electronic protocol for collection of clinical and surgical data on chronic venous insufficiency

**DOI:** 10.1590/1677-5449.190127

**Published:** 2020-07-31

**Authors:** Carla Contin Mottin, Henrique Jorge Stahlke, Osvaldo Malafaia

**Affiliations:** 1 Universidade Federal do Paraná – UFPR, Clínica Cirúrgica, Curitiba, PR, Brasil.

**Keywords:** chronic venous insufficiency, electronic protocols, vascular surgery

## Abstract

**Background:**

Use of electronic protocols for data collection and storage enables clinical research to be conducted dynamically, contributing to medical advances.

**Objectives:**

To create an electronic data base for collection of clinical and surgical data on chronic venous insufficiency (CVI), to facilitate production of scientific studies.

**Methods:**

Initially, a database was constructed by means of a bibliographic review of text books and relevant scientific articles for all vascular diseases and then a database on CVI was extracted. These data were computerized using the Integrated Electronic Protocols System (SINPE©) and then assessed in a pilot project.

**Results:**

The multidisciplinary electronic protocol for vascular diseases covered the following items: history taking, physical examination, work-up tests, types of treatment, and progression. Using these items, a master protocol was created containing 6,145 items, and then a CVI-specific protocol containing 2,877 items was compiled. The protocol’s functionality was tested in a pilot project, collecting data from medical records. The information collected was analyzed and illustrated graphically.

**Conclusions:**

It proved possible to create an electronic protocol for collection of clinical and surgical data on CVI. The protocol was incorporated into the SINPE©, greatly facilitating production of scientific research in the area.

## INTRODUCTION

The benefits of using IT resources in medicine have been irrefutably proven in the following situations: data capture and storage, production of scientific research, and distribution of medical literature.^1^^,^^2^

Studies with large numbers of patients guide changes in management of clinical cases, standardizing treatments, and improving the results obtained. This is the foundation of progress in medicine.^3^^-^5

Moreover, use of electronic patient records can improve interpretation and understanding of records of patient history, physical examinations, and diagnostic tests, in addition to providing rapid access to this information, facilitating production of scientific studies.^6^

Use of IT is also important for legal aspects, because of improved medical and laboratory record-keeping and significant reductions in medical prescription errors. Avoidable medical errors are responsible for more than 50,000 deaths per year in the United States. It will only be possible to reduce this alarming number by simultaneous adoption of several measures. However, one measure in particular did significantly reduce the number of errors in the medications administered to patients: substituting manual prescriptions for an electronic prescription system.^7^^-^9

Development of electronic protocols with the capacity for collection, structured storage, and processing of clinical data facilitates access and retrieval of this information. These protocols are therefore extremely useful tools for production of high-quality medical literature, when the objective is to expand production of prospective studies in shorter time frames.^10^

Many different electronic protocols have already been developed, focused on other diseases and in a variety of different branches of medicine.^11^^-^14 However, there are no similar publications for chronic venous insufficiency (CVI).

The multidisciplinary electronic protocol for CVI covers data from the patient history, including symptoms, risk factors, and lifestyle habits that affect development of the condition; describes in detail the important elements of the physical examination; presents the possible abnormal findings of work-up tests that lead to diagnosis of CVI; provides the clinical, etiology, anatomy, and pathophysiology classification (CEAP) and its scores, so that cases studied can be stratified; lists the different forms of treatment, ranging from clinical and surgical treatment through endovascular approaches; in addition to covering the important elements of disease progression after treatment. The objective of this study was to create an electronic protocol for clinical and surgical data collection focused on CVI, in order to support production of scientific studies of the disease.

## METHODS

Initially, a master protocol was created, entitled the “Multidisciplinary Vascular Diseases Protocol”, was subdivided into seven major areas: venous thromboembolism, chronic venous insufficiency, aneurysmal diseases, acute arterial occlusion, chronic arterial ischemia of upper limbs and supra-aortic trunks, chronic visceral ischemia, and chronic arterial ischemia of lower limbs.

Information on these different diseases was organized and input under the following headings: history taking, physical examination, work-up tests, diagnosis, treatment, and progression. Next, these items were imported to the Integrated Electronic Protocols System (SINPE© - Sistema Integrado de Protocolos Eletrônicos). This software program was developed by Prof. Dr. Osvaldo Malafaia, professor of Surgery at the Health Sciences Department of the Universidade Federal do Paraná (UFPR). Ownership of the intellectual property rights to this program were registered with the Brazilian patents and trademarks office (INPI - Instituto Nacional de Propriedade Industrial), run by the country’s ministry for industry, foreign trade and services, under registration number RS 06056-1, on February 17, 2009.

Once the items had been imported to SINPE, the entire content was available for viewing. The master protocol comprised 6,145 items and was used to generate a CVI-specific protocol containing 2,877 items.

The Multidisciplinary Electronic Protocol for Collection of Clinical and Surgical Data on Chronic Venous Insufficiency (MEPCCSD-CVI) is a descriptive study with methodology divided into five phases:

1. Creation of the theoretical foundation for clinical and surgical data on CVI by reviewing the specialized literature. Five text books were used for this process: Tratado de Flebologia e Linfologia (Treatise on Phlebology and Lymphology),^15^ Doenças Vasculares Periféricas (Peripheral Vascular Diseases),^16^ Vascular Surgery,^17^ Cirurgia Vascular: Cirurgia Endovascular – Angiologia (Vascular Surgery: Endovascular Surgery – Angiology),^18^ and Cirurgia Vascular (Vascular Surgery),^19^ in addition to relevant scientific articles;^20^^-^25
2. Computerization of the theoretical foundation data following the standard methodology of the “Computerized Protocols” research project run by the Postgraduate Program Surgery at the Health Sciences Department of the Universidade Federal do Paraná (UFPR). This research project incorporates the SINPE©, which is capable of storing and manipulating the data that comprises a theoretical foundation. The version used was developed in the C# software language, using Microsoft®.net Framework technology. This version offers user management, the capability for multi-center use, and support for manipulation of multimedia content. The program is distributed on CD-ROM. It can therefore be run on any computer, in different locations, as long as the minimum system requirements are met: Microsoft Windows 98® operating system, 32 megabytes of RAM memory, and a hard drive with at least 500 megabytes free space;
3. Application of the CVI-specific protocol using data from the patient records of people who underwent surgical treatment for lower limb varicose veins (International Classification of Diseases code I-83) provided by the vascular surgery service at the Hospital das Clínicas da Universidade Federal do Paraná (HC-UFPR) from 2000 to 2007. These data were collected in the form of a retrospective cross-sectional study of a series of 50 non-consecutive cases chosen at random. The inclusion criteria were: legible handwriting and complete descriptions of history taking, physical examination, surgery, and postoperative progression. The exclusion criteria were: indecipherable handwriting, missing details of symptoms from clinical history, failure to mention relevant risk factors, no CEAP classification, details missing from description of surgery, and failure to mention recovery or complications after treatment. Since the incidence of CVI is high, the number of cases analyzed was the minimum recommended by the information technology specialists who developed the SINPE© program. This pilot project was approved by the Human Research Ethics Committee and registered under registration number 2283.177/2010-07, with the objective of testing the functionality of the protocol. According to the ethics committee, there was no need to obtain free and informed consent;
4. Interpretation of the results obtained from the pilot project data collection employing the SINPE© Analyzer module, which offers rapid viewing of the information in the SINPE© electronic protocols. This module was used to plot several graphs illustrated in the results of the study;
5. Analysis of the results obtained from the pilot project data collection, presenting the incidence rates of certain items from patient histories, such as symptoms, lifestyle habits, elements of personal history listed as risk factors, elements of family history, abnormal color Doppler ultrasonography findings, CEAP classification and venous insufficiency score, types of surgical treatment performed, and postoperative progression. These analyses were illustrated in graphs, followed by explanations to aid in understanding them.

## RESULTS

The results were analyzed in two phases:

- phase 1: development of the MEPCCSD-CVI;
- phase 2: application of the MEPCCSD-CVI.

**Figure 1:**
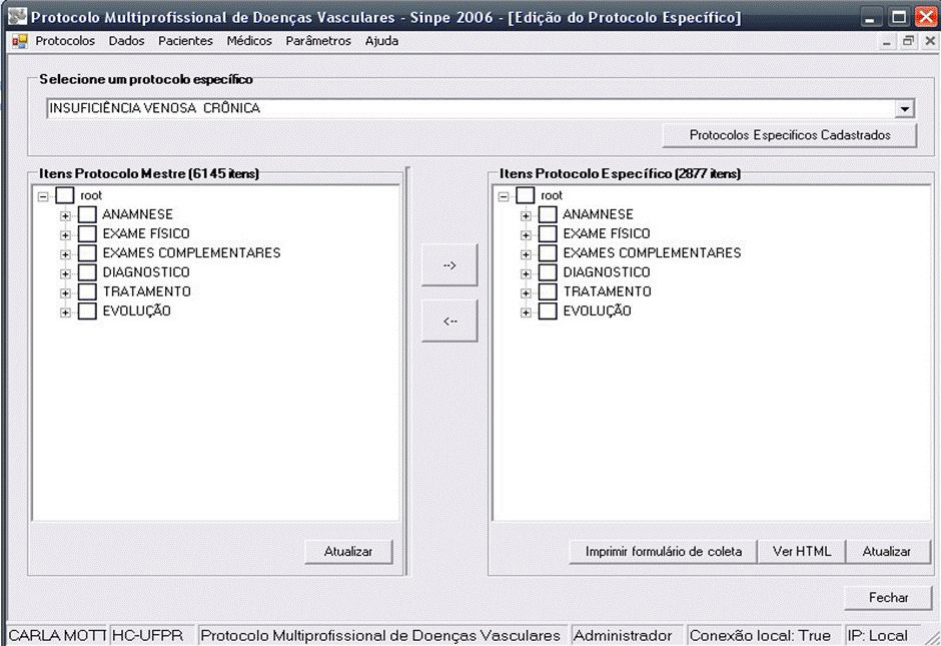
Specific protocol for chronic venous insufficiency.

In phase 1, results are shown as figures illustrating the screens displayed on the computer. The items cataloged were Patient history (Anamnese), Physical examination (Exame físico), Work-up tests (Exames complementares), Diagnosis (Diagnóstico), Treatment (Tratamento), and Progression (Evolução). The protocol comprised a total of 2,877 items (Figure 1).

In phase 2, a pilot project was conducted, registering patients on the protocol.

A total of 50 patient records were analyzed, from patients with CVI who underwent surgical treatment for varicose veins of the lower limbs, selected according to the criteria described in the methods section.

After registration of the patients, data on the specific items and subitems of the protocol were input from the patient medical records.

**Figure 2:**
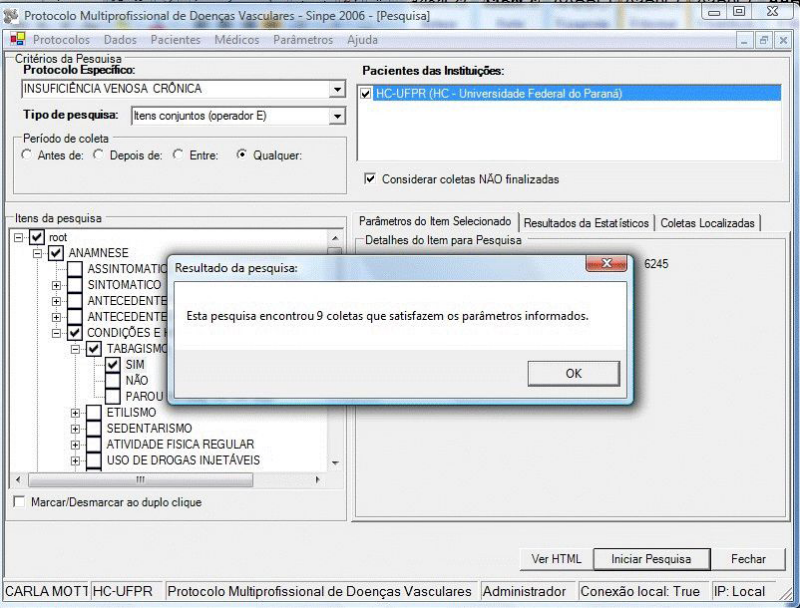
Example of the search screen for the parameter selected.

The study was then conducted. The results were displayed on the screen, showing the number of records located for each of the parameters chosen. the parameters of the item chosen, in this case smoking, can be observed in the example below. Nine records were located containing this item (Figure 2).

**Figure 3:**
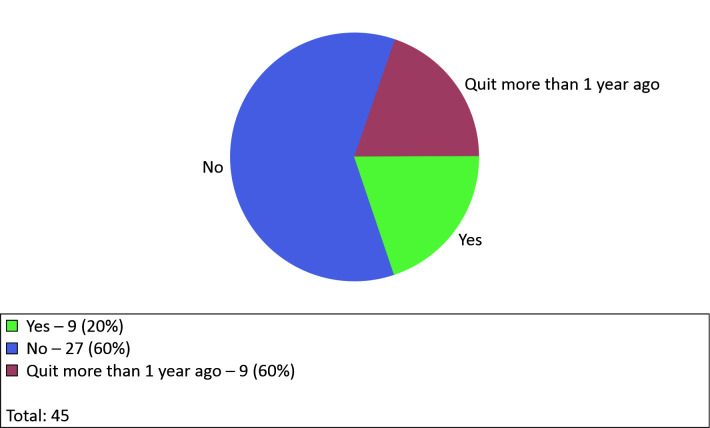
Example graph for the item selected: smoking.

The SINPE© Analyzer module was used to present the results of application of the MEPCCSD-CVI. This module analyzes the incidence of the items collected and plots graphs showing the results. For example, the item smoking is illustrated in the graph in Figure 3.

**Figure 4:**
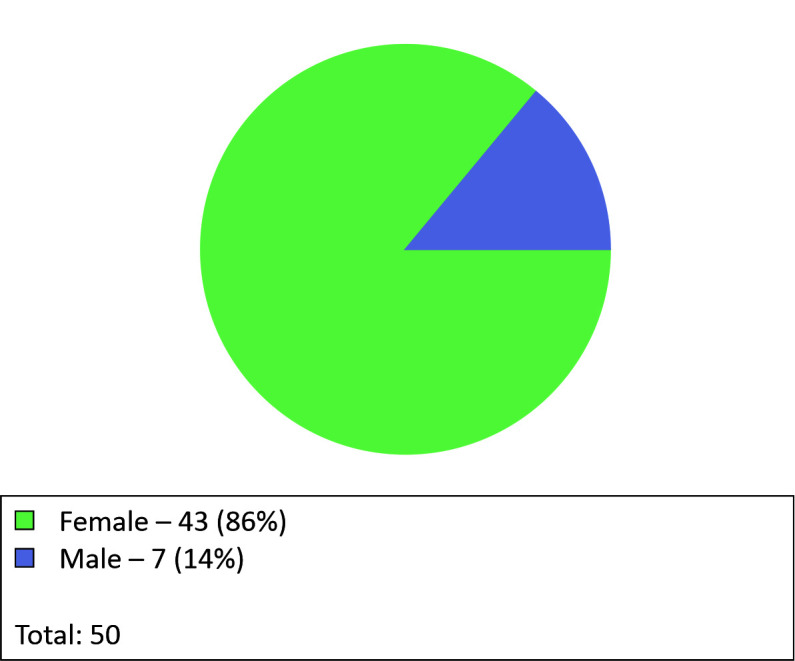
Graph for patients by sex.

**Figure 5:**
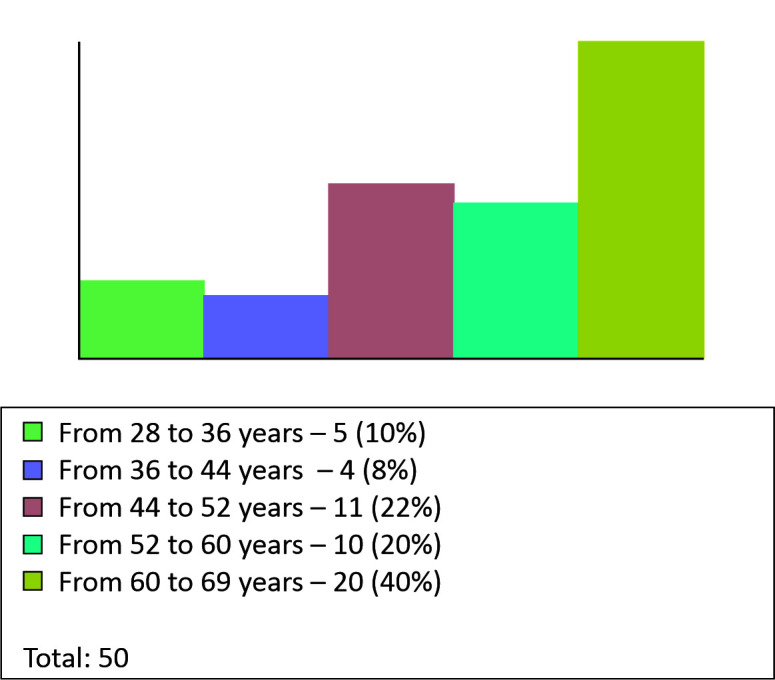
Graph for patients by age group.

In addition to smoking, several other items were identified and illustrated graphically. We observed that 43 patients (86%) were female and 7 (14%) were male (Figure 4). The mean age was 53 years, varying from 28 to 69 years (Figure 5).

**Figure 6:**
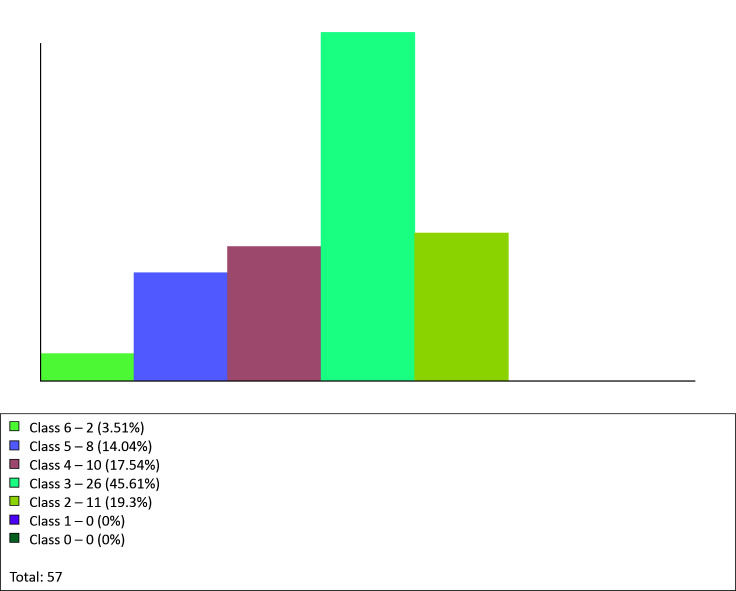
Graph for patients by CEAP clinical classification.

With regard to incidence of CEAP clinical classifications, the most prevalent was Class 3, with 26 records, accounting for 45.61% of cases, followed by Class 2, with 11 records, accounting for 19.3% of cases, and Class 4, with 10 records, accounting for 17.54% of cases. There were eight Class 5 records, accounting for 14.04% of cases, and just two Class 6 records, accounting for 3.51%. There were no records with CEAP Class 0 or Class 1. This graph illustrates a total of 57 items, because the program counts one record for patients who had the same classification for both limbs and two records for patients with different classifications for the left and right legs (Figure 6).

The types of surgical treatment performed were recorded separately for the great and small saphenous veins of the right and left lower limbs.

**Figure 7:**
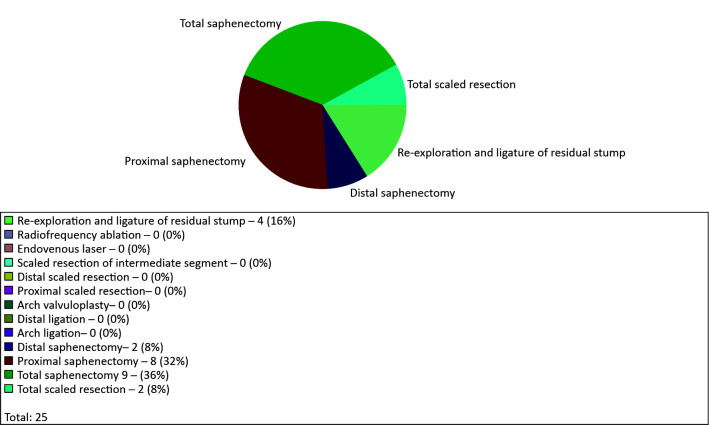
Graph for procedures performed on the right great saphenous vein.

Figure 7 illustrates the example of the right great saphenous vein:

- four cases of re-exploration and ligature of residual stump;
- two cases of total scaled resection;
- two cases of distal saphenectomy;
- eight cases of proximal saphenectomy; and
- nine cases of total saphenectomy.

Items on progression of cases evaluate postoperative progression in terms of presence or absence of complications, which complications occurred and improvement of symptoms.

**Figure 8:**
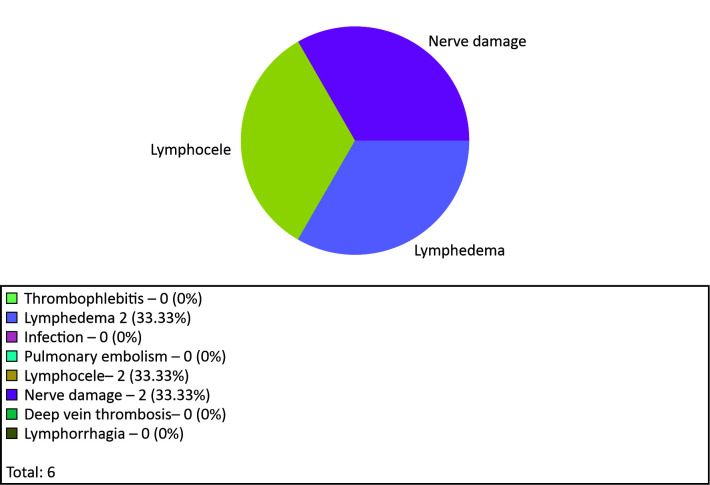
Graph for postoperative complications.

Complications observed on the seventh day after operating included two cases of lymphedema, two cases of lymphocele, and two cases of nerve damage (Figure 8).

## DISCUSSION

### Computerization of clinical data

Use of handwritten patient records for scientific research makes data collection much more difficult because, in general, these medical records are not filled out completely, are written by several different professionals, and may contain illegible handwriting. Additionally, extracting these data is very time-consuming. All of these factors are barriers to conducting high-quality research.^26^^,^^27^

Using electronic patient records offers countless advantages over manual patient records, including: reduced need for physical space and number of people to store documents; legibility of information; and ease of data storage and retrieval. Additionally, they can also provide support for multimedia resources, such as photographs, films, and digitalized examinations and test results. Another advantage is the fact that patients’ medical records can be accessed by several professionals simultaneously.^28^

Application of electronic protocols for data collection offers similar benefits to use of electronic patient records, with the advantage of standardization and hierarchization of the data collected. Electronic protocols increase the precision of records, enabling prospective and multicenter studies, in addition to increasing the accuracy of scientific research.^29^^-^31

However, use of the electronic protocol is in no way a substitute for patient medical records. The main difference between the two is that the protocol contains sources of information on a specific group of diseases, in contrast with patient medical records, which are specific to an individual patient and do not follow rigid completion criteria. They should continue to be filled out for follow-up and to provide a legal record of patient management. In common with research protocols, patient records are being moved over to electronic format with increasing frequency. The aim of this gradual change is to rationalize the time spent in medical consultations and facilitate retrieval of patients’ histories.^29^^-^31

### Construction of the MEPCCSD-CVI

Construction of the MEPCCSD-CVI started with extensive research in text books and scientific articles, correlating the items of greater importance.

This theoretical foundation was then computerized using SINPE©, which offers several tools for maintaining confidentiality and data protection. Differentiation of users and provision of different levels of authorization, the inability to alter a protocol (after one data collection has been conducted), and the inability to edit completed data collections are all features intended to prevent inadvertent changes to protocols.

### Application of the MEPCCSD-CVI

In order to assess the protocol’s functionality, it was applied to collection of data from the medical records of 50 patients with CVI who had undergone surgery for varicose veins. Limitations observed due to the fact that the analysis was retrospective included difficulties reading handwriting and missing information on history taking and physical examination on some patient medical records. The statistical significance of the data collected was not considered.

Data collected in the SINPE© are entered by mouse clicks. Although the process is objective and practical, it was necessary to train the data collector to ensure he took care with the items entered on the protocol, since, after each record was collected, it could not be edited.

The principles of navigating SINPE© are similar to those of Microsoft Windows®. It can be run over the internet and using handheld computers. These features were not tested in the pilot project, but they are very useful for prospective studies. There is also the option to print out the protocol for paper-based data-collection, if necessary.

The SINPE© Analyzer module was then used for statistical analysis of the data collected, identifying items collected and automatically plotting graphs. This module is very rapid and effective for use in scientific studies.

SINPE© has been approved by the health professionals who have used it, increasing scientific output and reducing the time spent on clinical trials by 50%. The current version allows protocols to be used via intranet or internet and to be updated on the system for data collection at any time, regardless of what institution is using the protocol.^32^

The objective of the MEPCCSD-CVI is to increase production of scientific research, since it offers security and uniformity for data storage, facilitating collection and analysis. It thus reduces the time taken to produce research and increases its credibility.

## CONCLUSIONS

The MEPCCSD-CVI was constructed from a theoretical foundation of clinical data relevant to the disease, input on the SINPE© computer program. Its functionality was tested by collecting data from patient medical records, which were then analyzed using the SINPE© Analyzer module. It is therefore concluded that the MEPCCSD-CVI is an excellent resource for data collection and storage, facilitating future research in the area.
