# Supplementary material for: Multidisciplinary electronic protocol for collection of clinical and surgical data on chronic venous insufficiency
Source: J Vasc Bras. 2020 Jul 31;19:e20190127. doi: 10.1590/1677-5449.190127 (PMC8202178; doi:10.1590/1677-5449.190127)
Supplement: Translation

# Translation: Protocolo eletrônico multiprofissional de coleta de dados clínicos e cirúrgicos em insuficiência venosa crônica

**Authors:** Carla Contin Mottin, Henrique Jorge Stahlke, Osvaldo Malafaia

**Affiliations:** 1 Universidade Federal do Paraná – UFPR, Clínica Cirúrgica, Curitiba, PR, Brasil.

**Keywords:** insuficiência venosa crônica, protocolos eletrônicos, cirurgia vascular

## Abstract

**Contexto:**

A utilização de protocolos eletrônicos para coleta e armazenamento de dados permite a realização de pesquisas clínicas de forma dinâmica, contribuindo com a evolução da medicina.

**Objetivos:**

Criar uma base eletrônica de coleta de dados clínicos e cirúrgicos referente à insuficiência venosa crônica (IVC) para auxiliar na realização de estudos científicos.

**Métodos:**

Inicialmente, criou-se uma base de dados de todas as doenças vasculares e, em seguida, de IVC, por meio da revisão bibliográfica de livros-texto e de artigos científicos relevantes. A informatização desses dados foi realizada pelo programa Sistema Integrado de Protocolos Eletrônicos (SINPE©) e avaliada por um projeto piloto.

**Resultados:**

O protocolo eletrônico multiprofissional de doenças vasculares abrangeu os itens anamnese, exame físico, exames complementares, tipos de tratamento e evolução. Com isso, criou-se um protocolo mestre contendo 6.145 itens, e após gerou-se um protocolo específico de IVC totalizando 2.877 itens. A funcionalidade do protocolo foi testada com um projeto piloto, coletando-se dados de prontuários. Realizou-se o cruzamento das informações coletadas, que foram demonstradas na forma de gráficos.

**Conclusões:**

Foi possível criar um protocolo eletrônico para coleta de dados clínicos e cirúrgicos referente à IVC. Esse protocolo foi incorporado ao SINPE©, facilitando imensamente a realização de pesquisas científicas nessa área.

## INTRODUÇÃO

Os benefícios da utilização de recursos de informática na medicina já foram amplamente comprovados para as seguintes situações: captura e armazenamento de dados, produção de pesquisas científicas, e distribuição da literatura médica^1^^,^^2^.

Os estudos com grande quantidade de pacientes norteiam as mudanças nas condutas dos casos clínicos, tornando os tratamentos uniformes e melhorando os resultados obtidos. Essa é a base para a evolução da medicina^3^^-^5.

Dessa maneira, o uso de prontuários eletrônicos promove melhor interpretação e compreensão dos registros de anamneses, exames físicos e exames diagnósticos; além disso, permite o acesso rápido a essas informações, facilitando o desenvolvimento de estudos^6^.

O uso da informática também é importante no aspecto legal, por meio da melhor estruturação dos registros médicos e laboratoriais, e na diminuição significativa dos erros de prescrições médicas. Erros médicos evitáveis são responsáveis por mais de 50.000 mortes por ano nos Estados Unidos. A redução desse número alarmante só é possível com a adoção simultânea de diversas medidas. Entretanto, uma medida isolada reduziu significativamente o número de erros em medicações aplicadas aos pacientes: mudança de prescrição manual para eletrônica^7^^-^9.

O desenvolvimento de protocolos eletrônicos com capacidade de realizar coleta, armazenamento estruturado e processamento de dados clínicos facilita o acesso e a recuperação dessas informações. Dessa forma, esses protocolos são ferramentas extremamente úteis na produção de literatura médica de qualidade, com o objetivo de ampliar a concretização de estudos prospectivos em menor tempo^10^.

Vários protocolos eletrônicos em relação a outras doenças, em diversas áreas da medicina, já foram desenvolvidos^11^^-^14. Porém, não há publicação semelhante para insuficiência venosa crônica (IVC).

O protocolo eletrônico multiprofissional em IVC relaciona dados referentes à anamnese, incluindo sintomas, fatores de risco e hábitos de vida que influenciam no desenvolvimento dessa afecção; descreve detalhadamente os elementos importantes no exame físico; apresenta as alterações possíveis nos exames complementares que levam ao diagnóstico de IVC; expõe a classificação clínica, etiologia, anatomia, fisiopatologia (CEAP) e sua pontuação, para que os casos estudados possam ser delimitados; relaciona as diferentes formas de tratamento, desde o tratamento clínico, cirúrgico até endovascular; além de incluir os itens importantes na evolução da doença após a terapêutica. Este estudo tem como objetivo, criar um protocolo eletrônico de coleta de dados clínicos e cirúrgicos referente à IVC para auxiliar na realização de estudos científicos nessa área.

## MÉTODOS

Primeiramente, foi criado um protocolo mestre, denominado “Protocolo Multiprofissional de Doenças Vasculares”, que incluiu sete grandes áreas: tromboembolismo venoso, insuficiência venosa crônica, doenças aneurismáticas, oclusão arterial aguda, isquemia arterial crônica de membros superiores e troncos supra-aórticos, isquemia visceral crônica e isquemia arterial crônica de membros inferiores.

As informações sobre essas diferentes doenças foram organizadas e inseridas nos itens anamnese, exame físico, exames complementares, diagnóstico, tratamento e evolução. Em seguida, esses itens foram introduzidos no programa Sistema Integrado de Protocolos Eletrônicos (SINPE©). Esse programa de computador foi criado pelo Prof. Dr. Osvaldo Malafaia, professor de Clínica Cirúrgica do Setor de Ciências da Saúde da Universidade Federal do Paraná (UFPR). A propriedade intelectual desse programa foi registrada no Instituto Nacional de Propriedade Industrial (INPI) do Ministério da Indústria, Comércio Exterior e Serviços do Brasil, sob o número RS 06056-1, em 17 de fevereiro de 2009.

Com a inserção dos itens mencionados no parágrafo anterior, foi possível a visualização de todo o conteúdo. O protocolo mestre totalizou 6.145 itens, com os quais foi possível gerar o protocolo específico de IVC, totalizando 2.877 itens.

O Protocolo Eletrônico Multiprofissional de Coleta de Dados Clínicos e Cirúrgicos em Insuficiência Venosa Crônica (PEMCDCC em IVC) é um estudo descritivo, e sua metodologia foi dividida em cinco fases:

1. Criação da base teórica de dados clínicos e cirúrgicos em IVC a partir de revisão de literatura especializada. Para tanto, cinco livros-texto foram utilizados: *Tratado de Flebologia e Linfologia* 15; *Doenças Vasculares Periféricas* 16; *Vascular Surgery* 17; *Cirurgia Vascular* 18 e *Cirurgia Vascular* 19, além de artigos científicos relevantes^20^^-^25;
2. Informatização da base teórica de dados seguindo a metodologia da linha de pesquisa denominada “Protocolos Informatizados”, do Programa de Pós-Graduação em Clinica Cirúrgica do Setor de Ciências da Saúde da Universidade Federal do Paraná (UFPR). Essa linha de pesquisa incorporou o SINPE©, que é capaz de armazenar e manipular os dados de uma base teórica. A versão utilizada foi desenvolvida em linguagem C# (C – Sharp), utilizando a tecnologia de programas da Microsoft® denominada .net Framework. Essa versão permite o gerenciamento dos usuários, a possibilidade de uso por ambiente multi-institucional, e manipulação de itens de multimídia. A distribuição do programa é feita através de CD-ROM. Dessa forma, pode ser manipulado de qualquer computador, em diferentes locais, desde que possua como configuração mínima: sistema operacional Microsoft Windows 98®, 32 *megabytes* de memória RAM e disco rígido com no mínimo 500 *megabytes* disponíveis;
3. Aplicação do protocolo específico de IVC com o uso de dados de prontuários de pacientes submetidos a tratamento cirúrgico de varizes de membros inferiores [Código Internacional de Doenças (CID) I-83] no Serviço de Cirurgia Vascular do Hospital das Clínicas da Universidade Federal do Paraná (HC-UFPR), entre os anos de 2000 e 2007. Esses dados foram coletados por meio de um estudo transversal retrospectivo de uma série de 50 casos não consecutivos, escolhidos aleatoriamente. Os critérios de inclusão foram: letra legível e descrição completa de anamnese, exame físico, descrição cirúrgica e evolução pós-operatória. Os critérios de exclusão foram: manuscrita indecifrável; ausência de detalhamento de sintomas na história clínica; não alusão sobre fatores de risco relevantes; falta de menção à classificação CEAP; falhas na descrição cirúrgica; e carência de citação à melhora ou a complicações apresentadas após o tratamento. Como a IVC apresenta uma grande incidência, o número de casos foi o mínimo indicado pelos especialistas em tecnologia da informação que desenvolveram o programa SINPE©. Esse projeto piloto foi aprovado pelo Comitê de Ética em Pesquisa em Seres Humanos (CEP) e registrado sob o número 2283.177/2010-07, com o objetivo de testar sua funcionalidade. E, de acordo com a orientação do CEP, não houve necessidade de aplicar a esses pacientes o termo de consentimento livre e esclarecido (TCLE);
4. Interpretação dos resultados obtidos pela coleta de dados do projeto piloto utilizando o módulo SINPE© Analisador, que permitiu a visualização rápida das informações presentes nos protocolos eletrônicos do SINPE©. Com a utilização desse módulo, foram criados vários gráficos ilustrados nos resultados deste trabalho;
5. Análise dos resultados obtidos pela coleta de dados do projeto piloto, apresentando as incidências de alguns itens de anamnese, como sintomas, condições de hábitos de vida, antecedentes pessoais relacionados como fatores de risco, antecedentes familiares, alterações evidenciadas no ecodoppler colorido, classificação CEAP e pontuação de insuficiência venosa, tipos de tratamento cirúrgico realizado e evolução pós-operatória. Essas avaliações foram demonstradas em forma de gráficos, seguidas de explicações para melhor compreensão destes.

## RESULTADOS

Os resultados foram analisados em duas fases:

- primeira fase: elaboração do PEMCDCC em IVC;
- segunda fase: aplicação do PEMCDCC em IVC.

Na primeira fase, os resultados foram demonstrados por figuras correspondentes às telas de apresentação no computador. Os itens catalogados foram Anamnese, Exame Físico, Exames Complementares, Diagnóstico, Tratamento e Evolução. Esse protocolo totalizou 2.877 itens (Figura 1).

**Figura 1:** Protocolo específico de insuficiência venosa crônica.

Na segunda fase, foi realizado o projeto piloto com cadastro dos pacientes nesse protocolo.

Foram avaliados os 50 prontuários de pacientes com IVC submetidos a tratamento cirúrgico de varizes de membros inferiores, selecionados de acordo com os critérios já descritos na metodologia do trabalho.

Após a inserção dos pacientes, realizou-se o preenchimento específico dos itens e subitens do protocolo visualizados nos prontuários médicos.

Posteriormente, a pesquisa iniciou. Os resultados apareciam na tela, contendo a quantidade de coletas encontradas em relação aos parâmetros informados. No exemplo a seguir, podemos observar os parâmetros do item selecionado, neste caso o tabagismo. Foram encontradas nove coletas em relação a esse item (Figura 2).

**Figura 2:** Exemplo de tela de pesquisa em relação ao parâmetro selecionado.

Para apresentação dos resultados da aplicação do PEMCDCC em IVC, utilizou-se o módulo SINPE© Analisador. Esse módulo analisa a incidência dos itens coletados e cria gráficos. Como exemplo, temos a seleção do item tabagismo demonstrado no gráfico a seguir (Figura 3).

**Figura 3:** Exemplo de gráfico do item selecionado: tabagismo.

Além do tabagismo, vários itens encontrados foram demonstrados em forma de gráficos. Observamos 43 pacientes (86%) do sexo feminino e 7 (14%) do sexo masculino (Figura 4). A idade média foi de 53 anos, variando de 28 a 69 anos (Figura 5).

**Figura 4:** Gráficos de pacientes em relação ao sexo.

**Figura 5:** Gráficos de pacientes em relação à faixa etária.

Com relação à incidência da classificação clínica CEAP, observou-se maior prevalência da Classe 3, com 26 coletas, indicando 45,61% dos casos; seguida da Classe 2, com 11 coletas, indicando 19,3% dos casos, e da Classe 4, com 10 coletas, indicando 17,54% dos casos. Na Classe 5, obteve-se oito coletas, indicando 14,04% dos casos; e na Classe 6, apenas duas coletas, indicando 3,51%. A Classe 0 e 1 não apresentaram nenhuma coleta. Nesse gráfico, o total de itens foi 57, pois o programa conta como uma coleta quando os pacientes apresentam a mesma classificação para os dois membros, e conta como duas coletas quando o paciente apresenta classificação diferente para os membros inferiores direito e esquerdo (Figura 6).

**Figura 6:** Gráfico de pacientes em relação à classificação clínica do CEAP.

Em relação aos tipos de tratamento cirúrgico realizados, foram demonstrados separadamente, de acordo com a veia safena magna e parva de membro inferior direito e esquerdo.

Como exemplo, temos a análise da veia safena magna direita (Figura 7):

**Figura 7:** Gráfico de procedimentos realizados na veia safena magna direita.

- quatro casos de reexploração e ligadura de coto residual;
- dois casos de ressecção escalonada total;
- dois casos de safenectomia distal;
- oito casos de safenectomia proximal; e
- nove casos de safenectomia total.

Os itens da evolução avaliaram o pós-operatório em relação à presença ou não de complicações, quais as complicações apresentadas e a melhora dos sintomas.

Entre as complicações encontradas no sétimo dia de pós-operatório, foram observados dois casos de linfedema, dois casos de linfocele, e dois casos de lesão de nervos (Figura 8).

**Figura 8:** Gráfico de complicações pós-operatórias.

## DISCUSSÃO

### Informatização dos dados clínicos

A utilização de prontuários manuscritos para realização de trabalhos científicos dificulta demasiadamente o levantamento de dados, pois, em geral, esses prontuários são preenchidos de forma incompleta, por diferentes profissionais, e podem conter escrita ilegível. Além disso, o levantamento desses dados é muito demorado. Todos esses fatores impedem a realização de pesquisas de qualidade^26^^,^^27^.

O emprego de prontuários eletrônicos apresenta inúmeras vantagens em relação ao prontuário manual, entre elas podemos citar: diminuição de espaço físico e do número de pessoas para armazenar documentos; legibilidade das informações; facilidade de recuperação e de arquivamento de dados. Além disso, permite o uso de recursos de multimídia, como fotos, filmes e digitalização de exames. Outra vantagem é o fato de que os prontuários podem ser acessados por vários profissionais simultaneamente^28^.

A aplicação de protocolos eletrônicos para coleta de informações apresenta benefícios similares aos do uso do prontuário eletrônico, com a vantagem da padronização e hierarquização dos dados obtidos. Os protocolos eletrônicos aumentam a exatidão dos registros, possibilitando a realização de trabalhos prospectivos e multicêntricos, além de ampliar a veracidade das pesquisas científicas^29^^-^31.

Entretanto, o uso de protocolo eletrônico não aparece de nenhuma forma como substituto do prontuário médico de pacientes. A maior diferença entre ambos é que o protocolo contém fontes de informações sobre um determinado grupo de doenças, ao contrário do prontuário, que se refere a um paciente individual e não segue critérios rígidos de preenchimento. Este deve continuar a ser preenchido pelo médico para acompanhamento e também para registro legal das condutas. Semelhante ao que ocorre com protocolos de pesquisa, os prontuários médicos cada vez mais tendem à informatização. Com essa gradual mudança, busca-se a racionalização do tempo de consulta médica e a maior facilidade de busca do histórico de pacientes^29^^-^31.

### Confecção do PEMCDCC em IVC

A confecção do PEMCDCC em IVC iniciou com pesquisa extensa em livros-texto e artigos científicos, correlacionando os itens de maior importância.

A informatização da base teórica foi realizada por meio do SINPE©, o qual apresenta várias ferramentas para manter a confidencialidade e a proteção dos dados inseridos. A distinção dos usuários, os diferentes tipos de autorização concedida para cada pessoa, a impossibilidade de adulteração do protocolo (após ter sido executada uma coleta de dados) e a impossibilidade de edição de coletas concluídas são recursos que visam impedir alterações inadvertidas nos protocolos.

### Aplicação do PEMCDCC em IVC

Para avaliar sua funcionalidade, o protocolo foi aplicado coletando-se informações de prontuários de 50 pacientes com IVC, submetidos a cirurgia de varizes. Como limitações, devido ao fato de a análise ter sido retrospectiva, observou-se dificuldade na legibilidade da escrita manual e falta de informações de anamnese e exame físico em alguns prontuários. Não foi levada em consideração a significância estatística dos dados coletados.

Para realizar as coletas pelo SINPE©, os dados do protocolo foram preenchidos com cliques do *mouse.* Apesar de as coletas serem objetivas e práticas, foi necessário treinamento do coletor para que ele fosse cuidadoso quanto aos elementos colocados no protocolo, pois, após o término das coletas, eles não puderam ser editados.

O princípio de navegação do SINPE© é similar ao do Microsoft Windows®. Pode ser executado por meio da internet e de computadores de mão. Esses recursos não foram testados no projeto piloto, porém, são muito úteis para trabalhos prospectivos. Também existe a possibilidade de se imprimir o protocolo e coletar os dados em papel, caso haja necessidade.

Em seguida, utilizou-se o módulo SINPE© Analisador para avaliação estatística dos dados coletados, verificando a ocorrência de coleta dos itens e gerando gráficos de modo automático. Esse módulo promove grande agilidade e eficiência para realização de trabalhos científicos.

O SINPE© tem sido aprovado pelos profissionais de saúde que o utilizam, aumentando a produção científica e diminuindo em 50% o tempo gasto nas pesquisas clínicas. Na sua atual versão, permite que os protocolos sejam utilizados em ambiente intranet ou extranet e atualizados no sistema a qualquer momento para coleta de dados, seja qual for a instituição que estiver utilizando o protocolo^32^.

O PEMCDCC em IVC tem como alvo o aumento na produção de trabalhos científicos, pois proporciona segurança e uniformidade no armazenamento de dados e facilita a sua captação e análise. Desse modo, reduz o tempo de realização das pesquisas e aumenta sua credibilidade.

## CONCLUSÃO

O PEMCDCC em IVC foi criado a partir de uma base teórica de dados clínicos relevantes para essa doença, inseridos no programa de computador SINPE©. Sua funcionalidade foi testada por meio da coleta de informações de prontuários, que foram analisados com a utilização do módulo SINPE© Analisador. Dessa forma, concluiu-se que o PEMCDCC em IVC provou ser um excelente recurso para captação e armazenamento de dados, facilitando futuras pesquisas nessa área.
